# Single-Molecule Dynamics and Discrimination between Hydrophilic and Hydrophobic Amino Acids in Peptides, through Controllable, Stepwise Translocation across Nanopores

**DOI:** 10.3390/polym10080885

**Published:** 2018-08-08

**Authors:** Alina Asandei, Isabela S. Dragomir, Giovanni Di Muccio, Mauro Chinappi, Yoonkyung Park, Tudor Luchian

**Affiliations:** 1 Interdisciplinary Research Department, Alexandru I. Cuza University, 700506 Iasi, Romania; alina.asandei@uaic.ro; 2 Department of Physics, Alexandru I. Cuza University, 700506 Iasi, Romania; isdragomir@yahoo.ro; 3 Department of Industrial Engineering, University of Rome Tor Vergata, Via del Politecnico 1, 00133 Rome, Italy; gio.dimuccio@gmail.com; 4 Department of Biomedical Science and Research Center for Proteinaceous Materials (RCPM), Chosun University, Gwangju 61452, Korea

**Keywords:** nanopore, peptide sensing, electrophysiology, single-molecule sequencing

## Abstract

In this work, we demonstrate the proof-of-concept of real-time discrimination between patches of hydrophilic and hydrophobic monomers in the primary structure of custom-engineered, macro-dipole-like peptides, at uni-molecular level. We employed single-molecule recordings to examine the ionic current through the α-hemolysin (α-HL) nanopore, when serine or isoleucine residues, flanked by segments of oppositely charged arginine and glutamic amino acids functioning as a voltage-dependent “molecular brake” on the peptide, were driven at controllable rates across the nanopore. The observed differences in the ionic currents blockades through the nanopore, visible at time resolutions corresponding to peptide threading through the α-HL’s constriction region, was explained by a simple model of the volumes of electrolyte excluded by either amino acid species, as groups of serine or isoleucine monomers transiently occupy the α-HL. To provide insights into the conditions ensuring optimal throughput of peptide readout through the nanopore, we probed the sidedness-dependence of peptide association to and dissociation from the electrically and geometrically asymmetric α-HL.

## 1. Introduction

In nanopore-based resistive pulse sensing, fluctuations of ionic current flowing through a voltage-biased nanopore provide useful information about the identity and physico-chemical properties of a temporarily residing or translocating analyte [1,2,3,4,5,6]. The system was successfully applied in a broad gamut of contexts, which started historically with detecting polynucleotides translocating through a nanopore [7,8,9,10], and eventually led to developing a rapid and inexpensive DNA sequencing technology [11]. Another useful application of nanopore technology targets proteomics, for which it has been proven successfully to detect and distinguish between various conformations of proteins and peptides [12,13,14,15], and potentially sequence peptides or proteins [16,17,18,19,20,21,22]. The latest objective is of considerable interest, as single molecule sequencing of peptides aims to become the tool of choice for identifying protein biomarkers and diagnosing in real-time the onset of various human diseases. Common protein sequencing methods are hindered by certain limitations; for instance, mass spectrometry and Edman degradation fail to readout the whole sequence information on larger proteins or peptides, are generally expensive, and require highly trained personnel and sophisticated infrastructure. Although the free translocation of single stranded DNA (ssDNA), RNA or unfolded peptides and proteins across nanopores determines measurable reductions in the ionic current whose magnitude and duration are sensitive to the primary sequence composition, the speed of translocation is one of the factors that precludes resolution of their constituent monomers [23], thus calling for various approaches to mitigate this problem [24,25,26,27,28,29,30,31,32,33]. Here, we describe an extension of a recent approach, whereby by flanking the residues to be distinguished by segments of oppositely charged amino acids, the applied transmembrane potential enhances the polypeptide capture rate by the α-HL nanopore, and simultaneously increases the peptide’s residence time in the nanopore [34,35,36], thus enabling discrimination between selected groups of three alanine, tryptophan and a combination of the two in the primary structure of polypeptides [37]. For the purpose of this work, we focused on a 30-amino-acid-long peptide, and our results indicate the possibility of discrimination between groups of three, selected polar amino acids (serine) and hydrophobic ones (isoleucine). The ability to provide real-time, high-fidelity readout of segments in such peptides is relevant in peptidomics, as secreted short peptides signals (2–21 amino acids) are deemed important in cell-cell communication, they coordinate and integrate cellular functions, and they perform a variety of functions within cells [38]. One advantage of the α-HL for such purposes lies in its restricted geometry, meaning that ionic current blockades corresponding to reversible peptide-α-HL interactions reflect events associated to a captured peptide in the unfolded conformation, which is a prerequisite for the subsequent primary sequence readout.

In efforts to explore the occurrence and consequences of the synergistic couplings between the electroosmotic, entropic and electrostatic interaction contributions to peptides capture and trafficking across the nanopore, with direct implications to the efficient capture of peptide from solution and subsequent sequence readout, we devised a series of experiments along the following lines: (i) peptides were added to the *trans* side, and their vectorial entry with either N- or C-moiety head-on, which expectedly modified the enthalpic contribution to the free energy of capture through attractive or repulsive electrostatic interactions with the negatively charged α-HL’s β-barrel, was studied; (ii) peptides were added to the *cis* side, and their vectorial entry into the largely neutral α-HL’s vestibule was studied; and (iii) for a transiently trapped peptide extending along the nanopore, the influence of peptide-nanopore electrostatic interactions inside the α-HL’s vestibule and β-barrel on the peptide dissociation from the nanopore was investigated. Overall, the system seems to provide the sensitivity required to identify specific groups of amino acids, based on their physical properties, and it serves as a good model system to advent short peptides sequencing via single-channel electrical recordings.

## 2. Materials and Methods

### 2.1. Chemicals and Reagents

The 30-aminoacid peptides Pe4 and Pe6, whose sequences were engineered as Ac–R_12_–(X)_6_–E_12_–NH_2_ (where X means either Isoleucine (I) for Pe4 or Serine (S) for Pe6), were synthesized and purified by Schafer-N ApS (Copenhagen, Denmark). The 1,2-diphytanoyl-*sn*-glycerophosphocholine (DPhPC) lipid was purchased from Avanti Polar Lipids (Alabaster, AL, USA) and other chemicals such as α-hemolysin (α-HL), potassium chloride (KCl), *n*-pentane, hexadecane, and buffer (HEPES) were obtained from Sigma-Aldrich (Darmstadt, Germany). The stock solutions of the peptides were prepared at concentrations of 1 mM each in distilled water, and were kept at −20 °C before and after use. All experiments were performed at a room temperature of ~23 °C.

### 2.2. Electrophysiology

The nanopore recording chamber consisted of two compartments (denoted by *cis* (grounded) and *trans*) separated by a 25-μm-thick Teflon film (Goodfellow, Malvern, MA, USA), having an aperture of about 120 μm in diameter for the bilayer lipid membrane formation. The Montal-Mueller technique was employed to obtain the lipid membrane bilayer [39]. Briefly, the dissolved lipids in pentane were spread out on the surface of the electrolyte solution and, after evaporation of pentane, a stable solventless bilayer structure was formed across the aperture punctured in the Teflon film, which was pretreated with ~1.5 μL of 1:10 hexadecane/pentane solution. Each compartment was filled with equal volumes of the 2 M KCl electrolyte solution buffered in 10 mM HEPES at pH = 7. About 1 μL of the α-hemolysin protein solution was released into the *cis* compartment from a monomeric stock solution made in 0.5 M KCl. Once the successful insertion of a single α-HL heptamer was achieved, and depending on the particular experiment, either Pe4 (Ac–(R)_12_–(I)_6_–(E)_12_–NH_2_) or Pe6 (Ac–(R)_12_–(S)_6_–(E)_12_–NH_2_) peptide was added to the *cis* or *trans* chamber. The fluctuations of the ionic current through the protein pore, recorded at various transmembrane voltages, were amplified with an Axopatch 200B instrument (Molecular Devices, San Jose, CA, USA), and low-pass filtered at 10 kHz. Data acquisition was performed with a NI PCI 6221, 16-bit card (National Instruments, Austin, TX, USA) at a sampling frequency of 50 kHz, within the LabVIEW 8.20 (National Instruments, Austin, TX, USA) graphical programming environment. A Faraday cage (Warner Instruments, Hamden, CT, USA), mechanically isolated with a vibration-free platform (BenchMate 2210, Warner Instruments, Hamden, CT, USA) was used to shield the experimental set-up from the environmental electrical and mechanical noise. Numerical analysis and graphic representation of the recorded data were done in Origin 6 (OriginLab, Northampton, MA, USA) and pClamp 6.03 (Axon Instruments, Union City, CA, USA) software. The statistical analysis on the frequency and duration of peptide-induced current fluctuations through a single α-HL protein, were analyzed within the statistics of exponentially distributed events, as previously described [40].

## 3. Results and Discussion

### 3.1. Steric- and Hydrophilic-Based Discrimination of Amino Acids at the Most Constricted Region of the Nanopore

**Figure 1:**
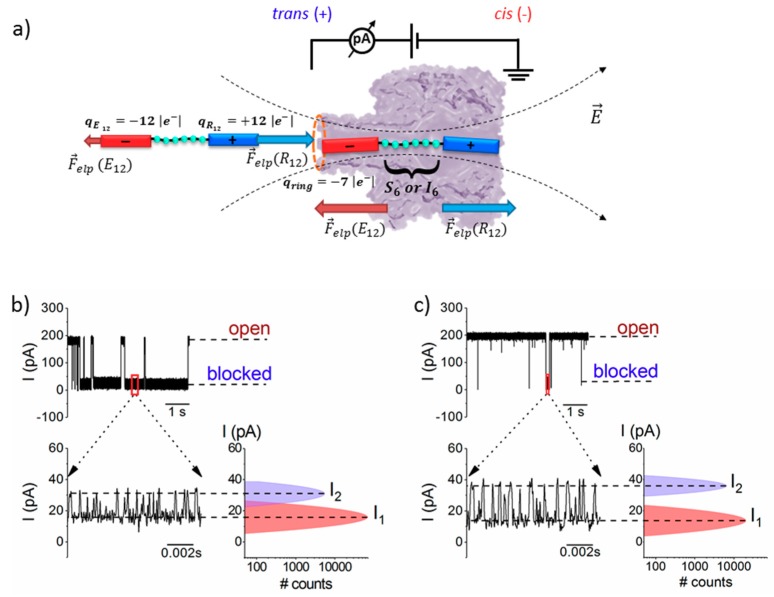
Schematic representation of the model resembling peptide interaction with the α-HL nanopore. (**a**) The R_12_ (bare charge ~ +12|e^−^|) and E_12_ (bare charge ~ −12|e^−^|) tails on the peptides, flanking a middle section containing either S_6_ or I_6_ amino acids are shown distinctly. As the peptide approaches the nanopore from the bulk, it experiences a stronger electric field ($\vec{E}$) near the nanopore’s opening [41], which results in uneven electrophoretic forces ($\vec{F}$_elp_) acting at the peptide’s oppositely charged extremities (at the shown +Δ*V*, the force acting on the R_12_ tail, $\vec{F}$_elp_(R_12_), is larger than the force acting on the E_12_ tail, $\vec{F}$_elp_(E_12_), thus driving peptide association to the nanopore). Once the peptide gets captured inside the nanopore, it assumes a meta-stable state, characterized to a first approximation (e.g., constant electric field across the nanopore from the applied Δ*V*) and at quasi-equilibrium conditions, by $\vec{F}$_elp_(R_12_) ≈ $\vec{F}$_elp_(E_12_). At neutral pH, the α-HL’s β-barrel entry is negatively charged (*q*_ring_ ~ −7|e^−^|). The selected original ionic current recordings illustrate the reversible blockades induced by the interactions of 20 µM *trans*-added S_6_-(Pe6) (**b**) and I_6_-containing peptide (Pe4) (**c**) with the α-HL, at Δ*V* = +100 mV. The representative zoomed-in segments below (**b**,**c**) show the ionic current fluctuations through the α-HL during a metastable capture of the corresponding peptides, and the corresponding ionic current amplitude histograms (see text).

To test the ability of α-HL to discriminate non-polar (isoleucine) and polar (serine) amino acid residues through the ionic current fluctuations pattern associated to the peptide reversibly blocking the nanopore, we engineered two types of peptides, to contain six isoleucine (I) or serine (S) residues, called Pe4 (the I_6_-containing peptide) and Pe6 (the S_6_-containing peptide), flanked by segments of oppositely charged amino acids (Figure 1). Once captured inside the nanopore by an applied transmembrane potential, either peptide gets trapped in a metastable state with the nanopore’s constriction region temporarily occupied by the peptide’s middle domain residues (Figure 1), which then constitute the main contributors to the recorded ionic current amplitude changes across the nanopore. In other words, the α-HL-peptide interactions at the nanopore’s constriction region, which augment the sensitivity of the targeted amino acids readout, were used to distinguish between isoleucine and serine residue, based on single-molecule electrophysiology-aided volumetric measurements.

**Figure 2:**
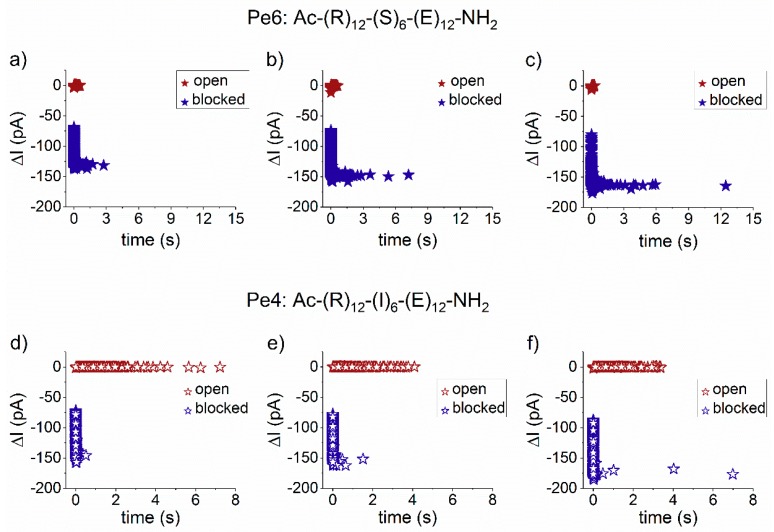
Scatter plots of dwell time vs. blockade amplitude (Δ*I*) of peptides-induced reversible obstructions of the ionic current through the α-HL nanopore. We display the scatter plot analysis corresponding to the Pe6 peptide interacting reversibly with the nanopore from the *trans* side, at distinct voltages: Δ*V* = +80 mV (**a**); Δ*V* = +90 mV (**b**); and Δ*V* = +100 mV (**c**). We present the scatter plot analysis of Pe4-α-HL interactions, at similar representative voltages: Δ*V* = +80 mV (**d**); Δ*V* = +90 mV (**e**); and Δ*V* = +100 mV (**f**). Distinctly drawn are the events corresponding to the open (red stars) and blocked sub-states (blue stars).

The scatter plots of dwell time vs. blockade amplitude of events shown in Figure 1, recorded at distinct positive transmembrane voltages, suggests that the transmembrane voltage affects distinctly the *trans*-added peptides interaction with the nanopore, with Pe6 being trapped longer than Pe4 peptide inside the α-HL, as the voltage increases (Figure 2). A more detailed analysis of this phenomenon is presented in the next paragraphs.

As shown in Figure 1b,c, there are two peaks in the current plot of fluctuations corresponding to the transiently nanopore-captured peptide, denoted by *I*_1_ and *I*_2_. Based on physical and geometrical considerations [37], we propose that these peaks are indicative of the back-and-forth journey of the middle region of the peptide across the constriction domain of the nanopore, under the net electric force experienced from contributions exerted at the ends of the peptide (vide infra).

By calculating the relative blockade extent corresponding to the residual ionic current (*I*_1_) ($\frac{\Delta I_{block,1}}{I_{open}}$, where $\Delta I_{block,1}=I_{blocked}-I_{open}$, $I_{blocked}$ is evaluated for the *I*_1_ blockade sub-state, and $I_{open}$ represents the ionic current measured through the free α-HL) from the current signatures found above for the two peptides occupying transiently the nanopore’s constriction region, resulted in $\frac{\Delta I_{block,1}}{I_{open}}$ (Pe6) = 0.91 ± 0.003 and $\frac{\Delta I_{block,1}}{I_{open}}$ (Pe4) = 0.93 ± 5.1 × 10^−4^. Following a similar route, the relative blockade extent corresponding to the residual ionic current (*I*_2_) was calculated at $\frac{\Delta I_{block,2}}{I_{open}}$ (Pe6) = 0.83 ± 0.002 and $\frac{\Delta I_{block,2}}{I_{open}}$ (Pe4) = 0.83 ± 0.004.

To account for the differences in the fractional blockades entailed by either peptide, and consistent with a previously described model [37], we posit that each blockade fluctuation reflects the reversible occlusion of the α-HL’s constriction region by a group of at least three serines (Pe6) or isoleucines (Pe4), respectively. We propose that, for either peptide, the deeper blockade assigned to the residual current (*I*_1_) reflects the instance when a group of three amino acids is centered on the constricting region of the nanopore, while the shallower one (*I*_2_) is consistent with the same group of resides shifting in and out of the constriction region, as the peptide at whole passes across the nanopore. Knowledge of microscopic details regarding the structure of α-HL, in conjunction with a rough volume-exclusion model for the ion current blockade through α-HL, allows a straightforward interpretation of such current fluctuations. On the one hand, the constriction region of the nanopore presents the highest sensitivity to resolving between closely sized amino acids, due to its volume (~924 Å^3^). On the other hand, the slightly higher extent of relative blockade for Pe4, calculated for the brief instance when a group of three amino acids is harbored on the constricting region of the nanopore ($\frac{\Delta I_{block,1}}{I_{open}}$), makes sense within the presented framework, as the less polar isoleucine present in its structure (individual volume in solution of ~175.4 Å^3^) is also bulkier than serine, present in Pe6 (individual volume in solution of ~100.7 Å^3^) [42].

**Figure 3:**
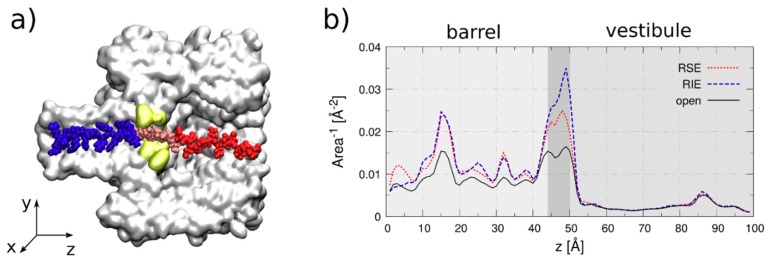
Atomistic simulations. (**a**) Snapshot of the system. Arginine tail is in blue, Glutamic acid in red while the neutral amino acid (Isoleucine, in this case) is in pink and it is in the pore constriction (yellow). Water, ions and lipid membrane are not represented for the sake of clarity. (**b**) The inverse of area available for the electrolyte passage for the open pore and for RIE (Pe4) and RSE (Pe6) cases.

To support these arguments, we performed all-atom molecular dynamics simulations. We setup the α-HL-membrane system following the same protocol employed in our previous work [37] and inspired by the early work of Aksimentiev and Schulten [43]. After α-HL-membrane equilibration, we independently equilibrated the peptide and merged the two systems. Details on the equilibration are reported in the Supplementary Materials. The peptide is imported into the pore using a constant velocity Steered Molecular Dynamics simulation. From this non-equilibrium translocation trajectory, we selected the conformation for which the central moiety of the peptide (isoleucine or serine) is in the pore constriction (see Figure 3a). Starting from this conformation, we performed a 32 ns equilibrium run with the amino acid close to the constriction constrained to its initial position along the pore axis. We then calculated, for each pore section z, the area A(z) available to the passage of the electrolyte (see Supplementary Materials). Figure 3b reports the inverse of the area for the open pore case (solid black), for the RIE (Pe4 peptide—dashed blue) and RSE (Pe6 peptide—fine dashed red). The quantity 1/A(z) is an indicator of the resistance that the ions find along their passage through the pore. For low electrolyte available area, A(z), 1/A(x) is large, as is the resistance to the ion passage. This is what happens in the pore constriction, 44 Å < z < 50 Å in Figure 3b, and, to a minor extent, in the β-barrel, 0 Å < z < 44 Å. The presence of the peptide clearly reduces the available area. It is apparent that this reduction is relevant only in the barrel and, in particular, in the constriction, while it is almost negligible in the vestibule, 50 Å < z < 100 Å. Interestingly, the peak in the constriction is significantly higher for RIE as expected from its larger size. Qualitatively similar results are obtained for different choices of the peptide conformation.

Despite its simplicity, the presented model, in which the ionic current blockade through nanopore is distinctly affected by the change in the electrical resistance brought about by an individual set of at least three amino acids that transiently diminish the nanopore’s constriction region free volume, may be useful for future efforts directed at α-HL-based peptide sequencing. At the same time, we accept that a more complex model considering other factors, such as amino acids partial (de)hydration at the constriction region, dynamic charge distributions and potential profile changes inside the nanopore due to residues passage, are needed to more accurately describe the blockade events as well as to correlate them properly with the nature of amino acids presented at the nanopore’s constriction region.

### 3.2. The Serine- and Isoleucine-Containing Peptides Interact Distinctly with the Nanopore, Despite Their Similar Net Charge

The peptide association rate constant to the α-HL was quantified through the inverse value of the average association times (${\hat{\tau }}_{on}^{-1}$) divided to the bulk concentration of the peptide ([peptide]) (*k_on_* = $\frac{{\hat{\tau }}_{on}^{-1}}{\left[\mathrm{peptide}\right]}$), whereas its dissociation rate constant was measured through the inverse value of the average dissociation times as *k_off_* = ${\hat{\tau }}_{off}^{-1}$.

**Figure 4:**
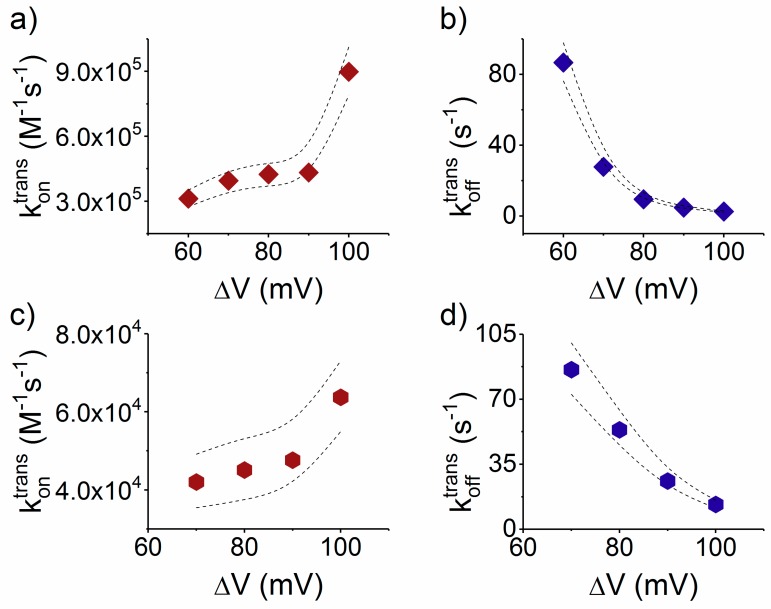
The *trans*-added, S_6_- and I_6_-containing peptides interact distinctly with the α-HL, despite their net charge and length. From the statistical analysis of the characteristic dwell-times resembling peptides association and dissociation from the nanopore, we calculated values of the voltage-dependent association (*k_on_*) and dissociation (*k_off_*) reaction rate constants of: S_6_-(Pe6) (**a**,**b**); and I_6_-(Pe4) (**c**,**d**). The dotted lines represent the upper and lower confidence limits mark the 95% confidence intervals for the estimated average values [40].

As we present in Figure 4a, the association rate constant for the *trans*-added, serine-containing peptide (Pe6) with the nanopore is almost one order of magnitude larger than that of the isoleucine-containing peptide (Pe4) (Figure 4c).

To explain this, we posit that the deformation and partial linearization of the approaching peptide by the electric field at the nanopore entrance on the *trans* side, as a preceding step to its successful threading into the narrow β-barrel lumen, is energetically more favorable for the more hydrophilic Pe6 peptide. It is not clear, a priori, the extent to which the hydrophilic/hydrophobic content in the primary structure of a peptide dominates its squeezing and subsequent entry into the narrow path of the nanopore. It is worth noting that in previous work authors have established that more hydrophobic peptides present a lower rate constant of association to the α-HL nanopore [44].

Opposite to the association process, we noted a slightly faster dissociation step from the nanopore of the more hydrophobic, isoleucine-containing Pe4 peptide (Figure 4d) as compared to the serine-containing one (Pe6) (Figure 4b). In accordance with the results from our group [45] and others [30,46,47], this finding fits in the scenario according to which non-specific electrostatic- and/or hydrogen bond-mediated interactions between the more polar peptide (Pe6), as compared to the more hydrophobic one (Pe4), and the nanopore’s inner wall, are major determinants of the overall friction experienced by moving peptide inside the nanopore, and contribute to a slowdown in the drift velocity of the Pe6 through the nanopore, relative to Pe4. Our findings complement others, emphasizing the requirement of hydrophobic binding sites to critically reduce the free energy barrier for translocating hydrophobic fragments of various polypeptides [48,49]. With possible benefits for the long term, these results reinforce the potential of the presented system for pinpointing the crucial contributions that govern biopolymers translocation through β-barrel proteins as a common scaffold used by protein-conducting channels, which is both ubiquitous and fundamental in many biological processes [50,51].

### 3.3. Sidedness-Dependence of Current Fluctuations Caused by Serine-Containing Peptides When Added from either Cis or Trans Side of the Nanopore

#### 3.3.1. The Case of Peptide Association to the Nanopore

The peptide capture is ruled by several concurrent effects, the most relevant being: (i) electrophoresis; (ii) entropic penalty due to the confinement; (iii) enthalpic contribution associated to specific interactions between the peptide and the pore entrance; and (iv) electroosmosis (elo).

**Figure 5:**
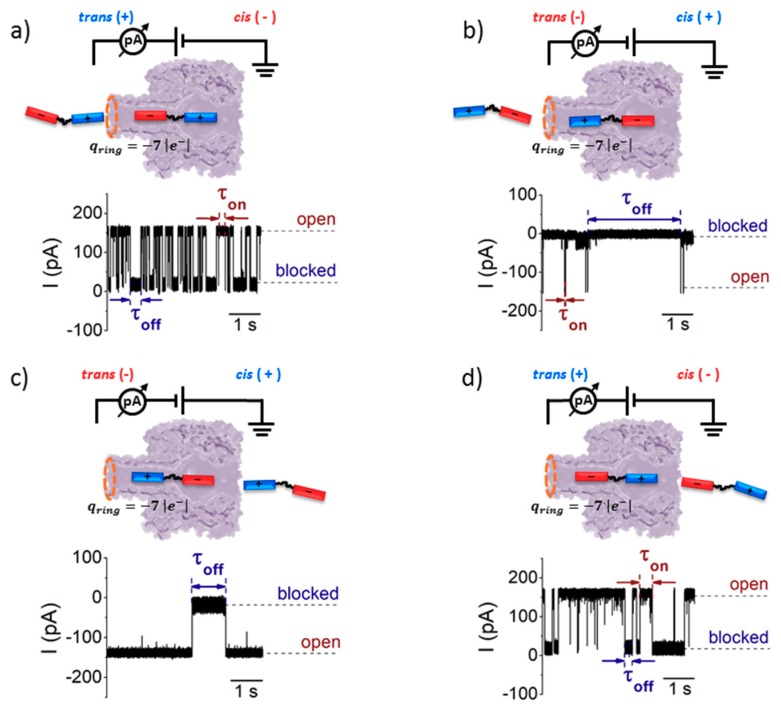
Sketch of the protocol aimed at studying the sidedness dependence of the S_6_-containing peptides-α-HL interactions. The peptide was added to the *trans* side of the membrane, and positive (**a**) or negative Δ*V*s (**b**) were employed to drive the peptide vectorially, with either the R_12_ or E_12_ moiety toward the nanopore’s β-barrel. The selected recordings below illustrate the details of current fluctuation through an α-HL inserted into a planar lipid bilayer, induced by the reversible peptide binding. The times between two consecutive peptide binding events (*τ_on_*) and of the peptide transient residence inside the nanopore (*τ_off_*) are also shown. (**c**,**d**) Precise similar chains of events are represented, except that the peptide was present on the *cis* side of the membrane. Note that, in this case, to drive the peptide inside the nanopore’s vestibule with the same orientation as above (i.e., R_12_ or E_12_ moiety head on), opposite polarities of the applied Δ*V*s, as compared to those used in (**a**,**b**), are needed.

As the heptameric α-HL nanopore is geometrically and electrically asymmetric, all four effects mentioned above alter distinctly the capture rate of entry of analytes on either side of the nanopore. Although the quantitative comparison among these four different effects is highly complex, some qualitative arguments allow unraveling the scenario emerging from our experiments. More specifically, based on the α-HL asymmetry, one would expect that:

- Electrophoresis is more intense at the trans, β-barrel mouth than at the vestibule entry of the nanopore. Indeed, as a first approximation, considering the nanopore and the membrane as perfect isolators, in stationary state, the electrical field streamlines moves only in the electrolyte. In a quasi-1D approximation of the pore, the electrical field flux E_z_A_z_, with E_z_ the component of the electrical field parallel to the pore axis and A_z_ the pore section, is constant along the pore. Hence, the electrical field is more intense in the narrower section of the pore. Consequently, the electrical field at the barrel mouth is larger than the one at the vestibule (see Supplementary Materials for physical details).
- Entropic penalty is larger on the trans side. In fact, the entropy cost of peptide squeezing inside the nanopore is larger for narrower pore sections.
- Due to the specific design of the studied peptides, which present opposite charges present at their ends, the enthalpy contribution to peptides capture depends on the sign of the applied voltage. At positive ΔVs, the trans-added peptide orients with the R_12_-containing moiety towards the negatively charged β-barrel α-HL’s opening. Consequently, the attractive electrostatic interactions manifested between the positively charged, R_12_-containing moiety of the peptide and the nanopore’s negatively charged β-barrel entry (at neutral pH, *q*_ring_ ~ −7|e^−^|) is expected to facilitate the peptide entry (Figure 5a). In contrast, at negative potentials, the trans-added peptides are driven with the negatively-charged, E_12_-containing moiety toward the β-barrel opening (Figure 5b), meaning that the peptide-nanopore electrostatic repulsions operate opposite to the electrophoretic force, and against peptide capture. On the other hand, the *cis*-added peptides are expected to associate to the nanopore with similar rates, regardless of the transmembrane potential polarity, as the vestibule entry of the nanopore is overall neutral at pH = 7 (Figure 5c,d), effectively nullifying the contribution of peptide–nanopore electrostatic interactions to the capture process.
- The electroosmotic flow (elo) favors peptide capture at negative potentials present on the peptide addition side. In such cases and judged from the peptide addition side perspective, the elo flow through the slightly anionic selective α-HL is directed toward the nanopore entry [33,43,52]. This implies that, for the *trans*-added peptides, electroosmosis favors the capture at negative Δ*V*s (i.e., the elo flow drives the peptide towards the nanopore) as compared to positive Δ*V*s (the elo flow drives the peptide away from the nanopore β-barrel and into the *trans* solution). Note however that, in the former situation (e.g., negative Δ*V*s), the electrophoretic force acting on the E_12_-containing moiety from peptides facilitate peptide migration toward the nanopore’s negatively charge β-barrel with the E_12_ tail head on, and this presents implications for the lumped force that determines peptides association to the nanopore (vide infra). The opposite occurs for *cis* added peptides, namely at negative Δ*V*s on the *trans* side, the elo flow drives the peptide away from the nanopore’s vestibule entry, while the elo flow elicited at positive Δ*V*s augment peptide association to the vestibule. As a side note, the elo flow is expected to be larger at the narrower, β-barrel section of the nanopore on the *trans* side (the mass flow rate in stationary state is constant, so that the smaller the cross-sectional area traversed by fluid, the higher the flow velocity).

For concreteness, we focused only on the serine-containing peptide. As shown in Figure 5, four different experimental combinations were envisioned, whereby the peptide is present on either the *cis* or *trans* side of the membrane, clamped at a positive or negative Δ*V*.

**Figure 6:**
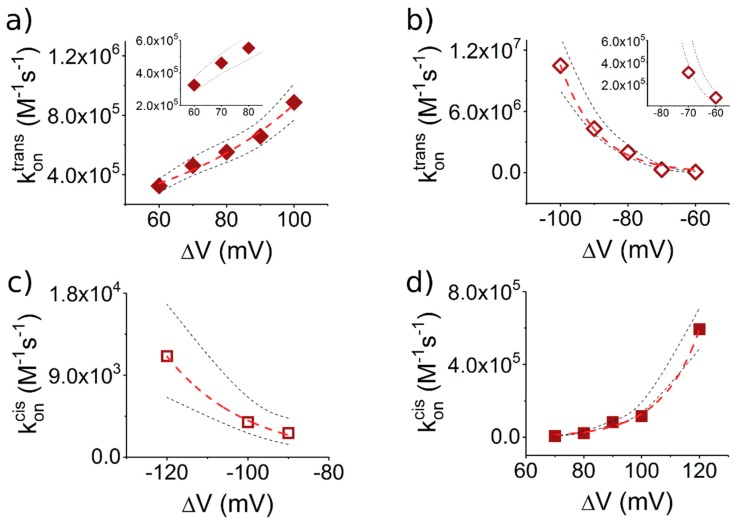
S_6_-containing peptides associate distinctly with the α-HL, depending of the polarity of the applied Δ*V* and the side on which they are added (*cis* or *trans*). All graphs display the voltage-dependent association rate constants (*k_on_*) of the Pe6 peptide (30 μM) with the α-HL. As in Figure 5: (**a**,**b**) the peptides were added to the *trans* side; and (**c**,**d**) the peptides were added on the *cis* side. The red dashed lines represent the best fit of the experimental data (open and filled rectangles) with the equation $k_{on}\;\left(\Delta V\right)=k_{on}\left(0\right)\exp\left(a\Delta V\right)$ (see text). The dotted lines represent the upper and lower limits of the 95% confidence intervals for the estimated average values [40].

Experimental data derived for association rates (*k_on_*, see Figure 6) indicate that, at high voltages (|Δ*V*| > 100 mV):

(1) $$k_{on}^{cis;-\Delta V}<k_{on}^{cis;+\Delta V}<k_{on}^{trans;+\Delta V}<k_{on}^{trans;-\Delta V}$$

The relations $k_{on}^{cis;-\Delta V}<k_{on}^{cis;+\Delta V}$ and $k_{on}^{trans;+\Delta V}<k_{on}^{trans;-\Delta V}$ can be reasonably explained in term of electroosmotic contribution (vide supra). Surprisingly, Equation (1) indicates that, despite the larger entropic cost, the association rates from the *trans* side are, in general, larger than peptide association from the *cis* side.

This counterintuitive effect can partially be explained with the larger electrophoretic contribution at the *trans* side. Another possible explanation relies on the fact that, for a polymer to enter the nanopore, it should be deformed first from its coiled configuration in solution—in which neither end of the polymer assumes the correct orientation for partitioning into the nanopore—to an extended-like chain, which promotes polymer insertion inside the nanopore. In the literature, this term refers to the free energy barrier for placing a polymer end at the nanopore mouth [53]. This loss in entropy of the polymer is further augmented once the polymer starts threading inside the narrow path of nanopore, as the range of allowed conformations on the polymer is reduced [53,54]. While the precise value of the entropic barrier depends on many factors, which themselves couple non-linearly, such as the polymer length, the ionic strength on the buffer, distribution of counterion cloud around the polymer, and the manifestation of the nanopore-polymer interactions, we posit that the locally larger electrical field at the *trans* entrance (compared to the *cis* entrance) assists more optimally the peptide deformation before the successful partitioning, on the *trans* side [55].

These observations are in apparent contrast with previous reports, where other proteins, polyelectrolytes or polynucleotide entry into the α-HL from the vestibule were suggested to be favored by a lower entropic barrier [56,57]. Thus, whether our observations are particular to heterogeneously charged (macro-dipole-like) peptides, which may fold differently from homogenously charged polymers, remain to be illuminated by further studies.

As a further step, and to account for the fact that, in the case of relatively low transmembrane potential values, the *trans*-added peptides associate faster to the nanopore at +Δ*V*s (Figure 6a, inset) than −Δ*V* s (Figure 6b, inset), we analyzed the association rates near equilibrium (Δ*V*→0). Data shown in Figure 6 are well described by the equation $k_{on}\;\left(\Delta V\right)=k_{on}\left(0\right)\exp\left(a\Delta V\right)$, where $k_{on}\;\left(0\right)$ represents that the association rate constant at Δ*V*→0. The resulting near equilibrium association constant are $k_{on}^{trans;+\Delta V}\left(0\right)=83.5\times 10^{3}$ ± $12.4\times 10^{3}$ (M^−1^s^−1^), $k_{on}^{trans;-\Delta V}\left(0\right)=1.2\times 10^{3}$ ± $0.6\times 10^{3}$ (M^−1^s^−1^), $k_{on}^{cis;+\Delta V}\left(0\right)=63$ ± 35.1 (M^−1^s^−1^) and $k_{on}^{cis;-\Delta V}\left(0\right)=26.1$ ± 8.9 (M^−1^s^−1^).

In other words, the following relations stand true:

(2) $$k_{on}^{cis;-\Delta V}\left(0\right)<k_{on}^{cis;+\Delta V}\left(0\right)<k_{on}^{trans;-\Delta V}\left(0\right)<k_{on}^{trans;+\Delta V}\left(0\right)$$

The relations above differ from the high voltage case (Equation (1)) only for the case of *trans*-added peptides, in which situation the positive Δ*V*s favors the capture as opposed to negative Δ*V*s. In accordance to the brief physical interpretation given above regarding the role of peptide–nanopore electrostatic interactions on the *trans* side, this result is not unexpected. That is, on the limit of vanishingly small Δ*V*s entailing the small contributions from the elo flow and electrophoretic force, positively biased nanopores on the *trans* side promote the proper peptide orientation and electrostatic attraction between the cationic (R_12_) tail from the peptide and the negatively charged β-barrel entry, which favors the peptides capture. Conversely, negatively biased nanopores on the *trans* side drive the peptide with the anionic (E_12_) tail toward the β-barrel entry, so that the ensuing electrostatic repulsion hinders the peptide association to the nanopore.

It therefore appears that, close to equilibrium, electrostatic interactions dominate the capture mechanism; however, we accept that this finding may not be complete, because hydrophobic as well as other hydrodynamic interactions may also to be involved in the process.

#### 3.3.2. The Case of Peptide Dissociation from the Nanopore

**Figure 7:**
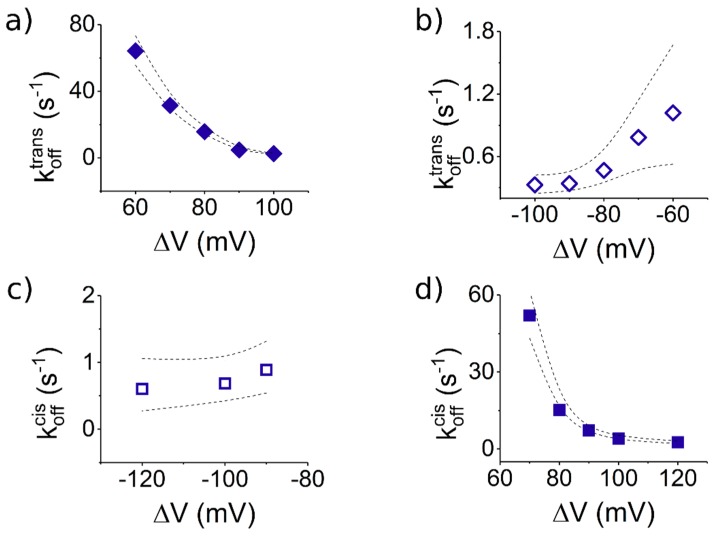
Dissociation rates of S_6_-containing peptides from the α-HL depend on the polarity of the applied Δ*V* and the side on which they are added (*cis* or *trans*). All the graphs display the voltage-dependent dissociation rate constants (*k_off_*) of the Pe6 peptide [30 μM] from the α-HL. As in Figure 5: (**a**,**b**) the peptides were added to the *trans* side; and (**c**,**d**) the peptides were added on the *cis* side. The dotted lines represent the upper and lower limits of the 95% confidence intervals for the estimated average values [40].

From the experiments undertaken with peptide added to the *trans* side of the membrane (Figure 5a,b), the statistical analysis of average times reflecting blockade duration of the nanopore by a single peptide (*τ_off_*), resulted in dissociation rate constants of the peptide from the nanopore (*k_off_*), which were larger at +Δ*V*s than at −Δ*V*s (Figure 7a,b).

To explain this finding, one can ignore to a first approximation the non-homogenous distribution of the electric field lines inside the nanopore [58], as the macro-dipole-like distribution of electric charge on the peptide structure will make it experience similar net electric forces during capture along the nanopore, regardless of the transmembrane voltage polarity. Instead, we favor a more plausible explanation based on the electrostatic interactions manifested between the charged β-barrel entry and the peptide’s moiety residing inside the β-barrel, during transient peptide entrapment inside the nanopore. As presented above, at +Δ*V*s, the *trans*-added peptide enters the nanopore with the R_12_ tail head-on. Thus, a trapped peptide under such circumstances, spanning the entire length of the nanopore, presents the E_12_ tail towards the negatively charged lumen entry (Figure 5a), and the ensuing lumen-E_12_ tail repulsive interactions favor peptide dissociation from the nanopore. In contrast, at −Δ*V*s, the *trans*-added peptide orients itself inside the nanopore with the R_12_ tail in the vicinity of the negatively charged lumen entry (Figure 5c), and the ensuing lumen-R_12_ tail attractive interactions stabilizes the peptide inside the nanopore. It should be kept in mind that such interactions manifest themselves especially when the peptide and the α-HL inner surface, respectively, are separated by sub-nanometer distances. Note that electrostatic interactions are screened by the counterions in the electrolyte, and the Debye screening length *κ*^−1^ ~ 1.9 Å at 2 M KCl and a temperature of 300 K (*κ*^−1^ = $\sqrt{\frac{\varepsilon_{r}\varepsilon_{0}k_{B}T_{m}}{2{\left|e^{-}\right|}^{2}N_{A}I1000}}$, where *ε_r_* and *ε*_0_ represent the relative permittivity of the electrolyte, ε*_r_* ~ 70, and vacuum permittivity, respectively; *N_A_* is Avogadro’s number; *e*^−^ is the elementary charge; *k_B_* is the Boltzmann constant; *T_m_* is the absolute temperature; *I* = $\frac{1}{2}\sum z_{i}^{2}C_{i}$ is the ionic strength of electrolyte; and *z_i_* and *C_i_* are the counterions valence number and their concentration in molar units), which is low relative to the average diameter of the α-HL’s β-barrel (~20 Å) or is vestibule (~46 Å). For our system, the average diameters of the peptide’s ends estimated with the Swiss-PdbViewer were ~13.2 Å (E_12_) and ~18 Å (R_12_); knowing that the diameter of the α-HL’s β-barrel is ~20 Å, it is conceivable that the peptide’s ends will still experience electrostatic interactions with the inner surface of the β-barrel, in agreement with previous findings on different systems [36,59].

We posit that the precise same physical and geometrical considerations account for the finding that a *cis*-added peptide captured transiently inside the nanopore (see the sketch in Figure 5c,d), dissociates faster at +Δ*V*s (Figure 7d) than −Δ*V*s (Figure 7c).

Notably, the peptide dissociation rate from the nanopore following its entry through either the *cis* or *trans* side, quantified under experimental conditions that ensure that peptide axial orientation is preserved with respect to the nanopore geometry while inside the nanopore (at +Δ*V*s, compare Figure 5a where the peptide enters from *trans* and Figure 5d where the peptide enters from *cis*, or, at −Δ*V*s, compare Figure 5b where the peptide enters from *trans* and Figure 5c where the peptide enters from *cis*), are within the same order of magnitude (compare data presented Figure 7a,d for the former case, at +Δ*V*s, and Figure 7b,c, for the latter case, at −Δ*V*s). This observation suggests that, for a nanopore-trapped peptide, its dissociation kinetics is largely independent upon the path it took to get there (i.e., entering either through the β-barrel or vestibule entry), as long as its vectorial orientation inside the nanopore relative to the axial geometry is preserved on either case.

## 4. Conclusions

In summary, the presented method demonstrates that α-HL represents a versatile single-molecule tool for identifying patches of polar and aliphatic residues in the primary structure of short peptides, via statistical analysis of ionic current fluctuations recorded during a peptide transit across the nanopore. In our experiments, the distributions of ionic fluctuations seen within individual peptide-induced α-HL conductance blockade events were interpreted as reflecting the stochastic transit of serine- or isoleucine-containing peptide across the constriction region of the α-HL. Such ionic fluctuations were characterized by two peaks in their amplitude histogram, which were found sensitive to the identity of a group of three serine or isoleucine amino acids. Depending on the charged state of the leading amino acids sequence from the studied peptide and considering the topological heterogeneity of the α-HL nanopore, the peptide capture by the nanopore and its residence inside it—which are both critical factors for high throughput and high signal-to-noise ratio sequencing—depend heavily on the transmembrane potential polarity and addition sidedness of the peptides. In short, configurations which promote attractive forces stemming from electrostatic interactions between the peptide with the α-HL’s β-barrel entry, and a narrower entry pathway for the peptide inside the nanopore, dramatically increase the peptide association rate to the nanopore, and the dwell-time of a captured peptide. For perspective, the presented approach is perfectly suited to tune the translocation speed of peptides through narrower nanopore systems, intended to provide increased spatial resolution (e.g., protein pores with engineered constriction regions matching in size the peptide bond length, or synthetic nanopores drilled in 2D materials such as graphene, WS_2_, and MoS_2_), and pave the way for peptide sequencing with single amino acid sensitivity. Although this proof-of-concept study rendered encouraging signs toward the goal of peptide sequencing, a precise understanding of how the force exerted on the trapped peptide influences it and alters flexibility and diffusion across the nanopore, and operating within a more complex model accounting for detailed positioning, hydration, charge distributions and binding affinity of amino acid residues to the nanopore, may be needed for the purpose of accurate peptide readout with nanopores.

## Supplementary Materials

The following are available online at http://www.mdpi.com/2073-4360/10/8/885/s1, S1. Quasi-1D expression of the electrical field, S2. Molecular dynamics simulation set-up.
