# Supplementary material for: Single-Molecule Dynamics and Discrimination between Hydrophilic and Hydrophobic Amino Acids in Peptides, through Controllable, Stepwise Translocation across Nanopores
Source: Polymers (Basel). 2018 Aug 8;10(8):885. doi: 10.3390/polym10080885 (PMC6403800; doi:10.3390/polym10080885)
Supplement: Supplementary file 1 [file polymers-10-00885-s001.zip › polymers-332587-SI.pdf]

Addendum

Supporting information for " Single-Molecule Dynamics and Discrimination between Hydrophilic and Hydrophobic Amino Acids in Peptides, through Controllable, Stepwise Translocation across Nanopores "

Alina Asandei[1,†], Isabela S. Dragomir [2,†] and Giovanni Di Muccio[3] and Mauro Chinappi[3] and Yoonkyung Park[4] and Tudor Luchian[2]

[1] Interdisciplinary Research Department, Department of Physics, Alexandru I. Cuza University, Iasi 700506, Romania
[2] Department of Physics, Alexandru I. Cuza University, Iasi 700506, Romania,
[3] Department of Industrial Engineering, University of Rome Tor Vergata, Via del Politecnico 1, 00133 Rome, Italy
[4] Department of Biomedical Science and Research Center for Proteinaceous Materials (RCPM), Chosun University, Gwangju, Korea.
* Correspondence: mauro.chinappi@uniroma2.it, y_k_park@chosun.ac.kr and luchian@uaic.ro
† These authors contributed equally to this work.

S1. Quasi-1D expression of the electrical field

In a continuum description, as a first approximation, since the dielectric constant of water is much larger that the one of the pore and of the membrane, we can treat the membrane as a perfect isolator, i.e. the electrical field is different from zero only in the electrolyte. Assuming that the electrolyte is neutral, the first Gauss equation reduces to

$$\int_S \mathbf{E} \cdot \hat{\mathbf{n}} \, dS = 0 \,, \tag{1}$$

where S is a closed surface and $\hat{\mathbf{n}}$ its normal.

In a pore, we can use a quasi-1D approximation, i.e., indicated with z the coordinate along the pore axis, we assume that i) the pore section $A(z)$ smoothly changes along z, ii) the only non negligible component of $\mathbf{E}$ is E_z and iii) that E_z it is constant on a given pore section, see figure 1. Using equation eq.(1) in the volume slide of size $\Delta z = z_2 - z_1$ (cyan shaded area in figure 1) and neglecting the contributions of the flux on pore walls we get

$$\int_S \mathbf{E} \cdot \hat{\mathbf{n}} dS = A(z_1)E_z(z_1) - A(z_2)E_z(z_2) = 0 \,, \tag{2}$$

i.e.

$$A(z_1)E_z(z_1) = A(z_2)E_z(z_2) = const \,. \tag{3}$$

Hence, for a pore or given section profile $A(z)$, the electrical field is more intense in the narrower sections.

Another route to show that E_z is larger in the narrower sections is to consider the differential form of the Ohm law

$$\mathbf{J} = \sigma \mathbf{E} \tag{4}$$

where $\mathbf{J}$ is the current density and σ the electrolyte conductivity. Indeed, in the quasi 1D approximation ($\mathbf{J} \simeq (0, 0, J_z)$, with J_z constant on the pore section), for each section of the pore, we have

$$J_z A(z) = I \tag{5}$$

with

$$I = \frac{\Delta V}{R_p} \tag{6}$$

the electric current, ΔV is the applied voltage and

$$R_p = \int_0^L \frac{1}{\sigma A(z)}\, dz \tag{7}$$

is the total resistance of the pore with L its length. Hence, from equations (4) and (5), we get

$$E_z(z) = \frac{I}{\sigma A(z)} \tag{8}$$

that, again, shows that the electrical field is more intense in the pore narrower sections.

The above arguments can be generalized adding a model for the access resistances as done, for instance, in [1,2] In this case, equations (5) and (8) still hold, while the eq. (6) reads

$$I = \frac{\Delta V}{R_p + R_{cis} + R_{trans}} \tag{9}$$

with R_{cis} and R_{trans} the pore access resistances at the cis and the trans side. In the literature, different models have been proposed for access resistance. If, following Wanunu et al. [1], we assumed that the pore mouths can be treated as semi-spherical electrodes, we have

$$R_{cis} = \frac{1}{\pi \sigma d_{cis}} \tag{10}$$

and

$$R_{trans} = \frac{1}{\pi \sigma d_{trans}}\,, \tag{11}$$

with d_{cis} and d_{trans} the diameters of cis and trans entrances. In this approximation, the current density $\mathbf{J}$ and the electrical field $\mathbf{E}$ outside the pore are radial and constant along an semi-spherical surface. Hence, indicated as J_r and E_r their intensities, the electrical current I is obtained as the current density J_r times the area of the semi-sphere of radius r. At the cis side, we obtained

$$I = 2\pi r_{cis}^2 J_{r,cis} \tag{12}$$

and

$$E_{r,cis} = \frac{I}{2\pi\sigma r_{cis}^2}\,, \tag{13}$$

while at trans side

$$J_{r,trans} = \frac{I}{2\pi r_{trans}^2}\quad , \quad E_{r,trans} = \frac{I}{2\pi\sigma r_{trans}^2}\,, \tag{14}$$

where r_{cis} and r_{trans} are the distances from the center of the pore mouths, see figure 1.

Equation (13) and (14) clearly show that, far from the pore, the electrical field intensity depends only on the distance r_{cis} (or r_{trans}). Hence, far from the pore, no difference in the electrophoretic contribution between the cis and the trans side is expected. Instead, into the pore, the narrower

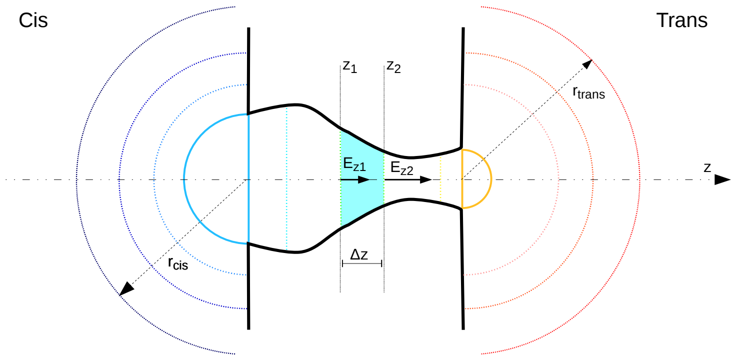

Figure 1. Sketch of the quasi-1D model. The only non negligible component of the electrical field **E** inside the pore is along the pore axis. Colored dashed lines are isolines of the electrical potential V (hence, **E** is normal to those lines). Outside the pore, a far field model, indicates that the electrical field is radial. The matching of the two solutions, quasi-1D inside the pore and semi-spherical outside, just outside the pore (solid semi-circles) has a certain degree of arbitrariness, see [2].

sections correspond to larger E, as demonstrated in eq. (5) and (8) and, hence, in the α-HL case, the electrical field at the barrel entrance is expected to be larger than the field at the vestibule entrance.

As a final comment, we would like to stress that, for a cylindrical pores of length L and diameter d, the equation (7) reduces to

$$R_p = \frac{4L}{\pi d^2 \sigma}, \tag{15}$$

that, combined with (10) and (11) gives

$$I = \frac{\Delta V}{\left(\frac{4L}{\pi \sigma d^2} + \frac{2}{\pi \sigma d}\right)}, \tag{16}$$

that, after some algebra, is identical to the result discussed in appendix of supporting information of [1] and in [2].

S2. Molecular dynamics simulation set-up

The membrane-αHL system has been built using the same protocol used in our previous works [3,4] that follows the one reported in [3,5,6]. In brief, the system was assembled starting from the αHL structure PDB_ID: 7AHL [7] taken from the OPM database [8]. The lipid membrane (POPC), the water molecules, and the ions to neutralize the system were added using VMD [9]. Then, the system is minimized and a 60 ps NVT simulation (time step 0.2 fs) was run with external forces applied to the water molecules to avoid their penetration into the membrane. Lipid heads have been constrained to their initial position by means of harmonic springs (spring constant $k = 1 kcal/(mol^2)$) acting on the phosphorus. A second equilibration run (1 ns NPT flexible cell, time step 1f̃s) was performed to compact the membrane. During this second run, the lipid heads were unconstrained. The third, and last, equilibration step consists of a NPT constant area simulation (2 ns, time step 2 fs) where all the atoms are unconstrained and no external forces act on the water molecules. The resulting triperiodic box, after the equilibration, has $L_x = 127.5$ Å, $L_y = 127.1$ Å, and $L_z = 180.0$ Å, and the total number of atoms is ~ 302000. Initial configurations of peptides are generated by using the PEPFOLD server [10] and then separately equilibrated in a triperiodic water box. Then, the two systems were merged, ions ($2M$ KCl) were added using VMD, and a short NPT equilibration is performed (2 ns, constant area NPT) until L_z reaches a stationary value. The resulting box has dimensions $L_x = 127.5$ Å, $L_y = 127.1$ Å (i.e.

the same as the original equilibrated αHL-membrane box) while $L_z \simeq 186.2$ Å (slightly different values are get for each peptide) and the overall number of atom is $\simeq 308000$.

A Steered Molecular Dynamics simulations was employed to bring the peptides at the pore's lumen entrance (trans side) and, then, into the pore. In particular, the peptide N-terminus was placed at ~ 15 Å from the αHL's trans entrance and then pulled inside the nanopore using a constant velocity Steered Molecular Dynamics (SMD) simulation at pulling speed $v_{SMD} = 0.025$Å$/$ps.

We selected the conformation for which the residues 15 and 16, i.e. the third and fourth residue of the neutral central region of the peptide, are closer to the pore constriction and used this configuration for a 32 ns equilibrium run where a spring in the z direction is applied to the Cα of the residue closer to the pore constriction (spring constant $k = 1kcal/(mol^2)$).

The simulations were performed using the NAMD software [11]. The CHARMM36 force field [12] was employed to model lipid, protein, and TIP3P water molecules [13]. NBFIX corrections were applied for ions [14].

Estimation of $A(z)$. The protocol for the estimation of the volume available to the electrolyte transport is the same employed in [15]. As a first step, we divided the system in cubic cells of size $\Delta x = \Delta y = \Delta z = 1$Å, and, for each frame, we used the VMD Volmap plug-in [9] to compute the occupancy map of the electrolyte, $m_{x,y,z}$, where x, y, z indicate the cell, $m_{x,y,z} = 1$ if the cells is within a Van der Waals radius of at least one water or ion atoms and $m_{x,y,z} = 0$ elsewhere. Then, we averaged $m_{x,y,z}$ over all frames and normalized it with the bulk value, the resulting averaged and normalized occupancy map is indicated with $M_{x,y,z}$. As already discussed in Aksimentiev and Schulten [5], "electrolyte pockets" are present close to constriction, see figure S2. The pockets do not contribute to the ion current. To filter out these pockets, we defined a trans→cis available channel as the pore region accessible to the electrolyte when moving from the barrel entrance towards the vestibule. This procedure excludes reentrant pockets directed towards the trans side. The same procedure is applied to get a cis→trans accessible pore, and the final occupancy map $\tilde{M}_{x,y,z}$ is obtained as the intersection of the trans→cis and cis→trans accessible pores. The occupancy map $\tilde{M}_{x,y,z}$ allows a direct estimation of the pore section $A(z)$ that can be calculated summing $\tilde{M}_{x,y,z}$ on slices of width Δz normal to the pore axis, in formula

$$A_z = \sum_{x,y} \tilde{M}_{x,y,z}^{x,y,z} \Delta x \Delta y \,. \tag{17}$$

References

1. Wanunu, M.; Morrison, W.; Rabin, Y.; Grosberg, A.Y.; Meller, A. Electrostatic focusing of unlabelled DNA into nanoscale pores using a salt gradient. *Nature nanotechnology* **2009**, *5*, 160–165.
2. Chinappi, M.; Luchian, T.; Cecconi, F. Nanopore tweezers: Voltage-controlled trapping and releasing of analytes. *Physical Review E* **2015**, *92*, 032714.
3. Bonome, E.L.; Cecconi, F.; Chinappi, M. Electroosmotic flow through and α-hemolysin nanopore. *Microfluidics and Nanofluidics* **2017**, *21*, 96.
4. Asandei, A.; Rossini, A.E.; Chinappi, M.; Park, Y.; Luchian, T. Protein nanopore-based discrimination between selected neutral amino acids from polypeptides. *Langmuir* **2017**, *33*, 14451–14459.
5. Aksimentiev, A.; Schulten, K. Imaging α-hemolysin with molecular dynamics: ionic conductance, osmotic permeability, and the electrostatic potential map. *Biophysical journal* **2005**, *88*, 3745–3761.
6. Comer, J.R.; Wells, D.B.; Aksimentiev, A. Modeling nanopores for sequencing DNA. In *DNA Nanotechnology*; Springer, 2011; pp. 317–358.
7. Song, L.; Hobaugh, M.R.; Shustak, C.; Cheley, S.; Bayley, H.; Gouaux, J.E.; others. Structure of staphylococcal alpha-hemolysin, a heptameric transmembrane pore. *Science* **1996**, *274*, 1859–1865.
8. Lomize, M.A.; Lomize, A.L.; Pogozheva, I.D.; Mosberg, H.I. OPM: orientations of proteins in membranes database. *Bioinformatics* **2006**, *22*, 623–625.

9. Humphrey, W.; Dalke, A.; Schulten, K.; others. VMD: visual molecular dynamics. *Journal of molecular graphics* **1996**, *14*, 33–38.
10. Thevenet, P.; Shen, Y.; Maupetit, J.; Guyon, F.; Derreumaux, P.; Tuffery, P. PEP-FOLD: an updated de novo structure prediction server for both linear and disulfide bonded cyclic peptides. *Nucleic acids research* **2012**, *40*, W288–W293.
11. Phillips, J.C.; Braun, R.; Wang, W.; Gumbart, J.; Tajkhorshid, E.; Villa, E.; Chipot, C.; Skeel, R.D.; Kale, L.; Schulten, K. Scalable molecular dynamics with NAMD. *Journal of computational chemistry* **2005**, *26*, 1781–1802.
12. Brooks, B.R.; Brooks III, C.L.; Mackerell Jr, A.D.; Nilsson, L.; Petrella, R.J.; Roux, B.; Won, Y.; Archontis, G.; Bartels, C.; Boresch, S.; others. CHARMM: the biomolecular simulation program. *Journal of computational chemistry* **2009**, *30*, 1545–1614.
13. Jorgensen, W.L.; Chandrasekhar, J.; Madura, J.D.; Impey, R.W.; Klein, M.L. Comparison of simple potential functions for simulating liquid water. *The Journal of chemical physics* **1983**, *79*, 926–935.
14. Luo, Y.; Roux, B. Simulation of osmotic pressure in concentrated aqueous salt solutions. *J.Phys. Chem. Lett.* **2009**, *1*, 183–189.
15. Di Muccio, G.; Rossini, A.E.; Di Marino, D.; Giuseppe, Z.; Chinappi, M. Computational assessment of peptide sequencing capability of a nanopore sensor. *in preparation*.
